# Supplementary material for: Marek’s Disease Virus Infection of Natural Killer Cells
Source: Microorganisms. 2019 Nov 20;7(12):588. doi: 10.3390/microorganisms7120588 (PMC6956363; doi:10.3390/microorganisms7120588)
Supplement: Supplementary file 1 [file microorganisms-07-00588-s001.pdf]

microorganisms

MDPI

Supplementary Material

Marek's Disease Virus Infection of Natural Killer Cells

Luca D. Bertzbach, Daphne A. van Haarlem, Sonja Härtle, Benedikt B. Kaufer and Christine A. Jansen

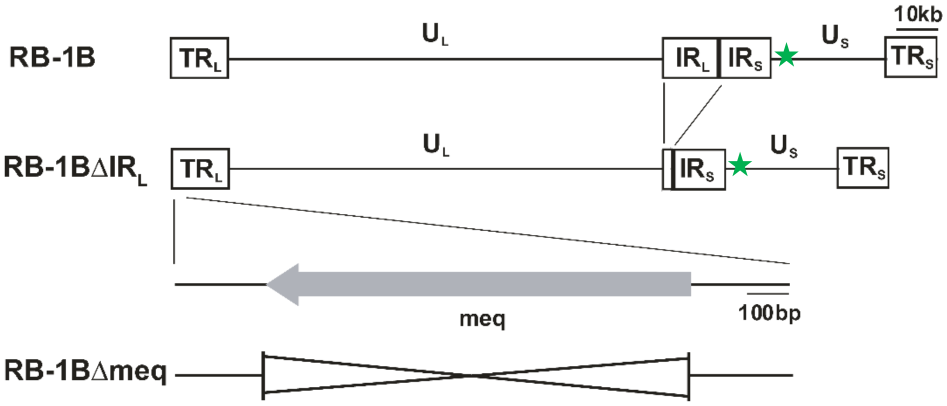

Figure S1. Generation of recombinant viruses. Schematic representation of the Marek's disease virus (MDV) genome with two unique regions, unique long (U_L) and short (U_S) that are flanked by terminal (TR_L and TR_S) and internal (IR_L and IR_S) inverted repeat regions. The focus is on the TR_L region that contains *meq*. The green asterisks indicate the location of the minimal fertility factor replicon (mini-F) in the U_S region.

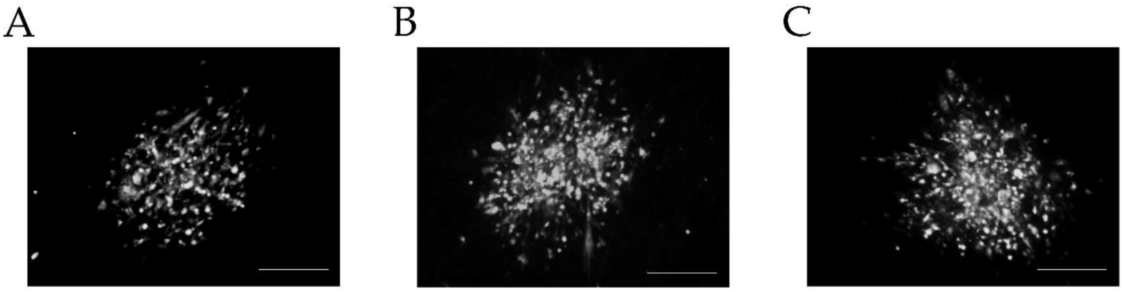

Figure S2. Representative plaque images. Plaque images from (**A**) RB-1B-infected CEC, (**B**) CVI-infected CEC and (**C**) RB-1BΔmeq-infected CEC. The scale bar corresponds to 100μM.

Table S1. Primers and TaqMan probes used in this study.

Construct	Sequence 5′ – 3′
ep[1] PCR forward primer (eGFP insertion)	GGTGACACGCGCGGCCTCGAACACAGCTGCAGGCCATGGTGA GCAAGGGCGAGG
ep PCR reverse primer (eGFP insertion)	CGTCGACCCGGGTACCTCTAGATCCGCTAGCGCTTTA CTTGTACAGCTCGTCCATGCC
ep PCR forward primer (Δ*meq*)	CAGGGTCTCCCGTCACCTGGAAACCACCAGACCGTAGACTGGG GGGACGGATCGTCAGCGGTAGGGATAACAGGGTAATCGATTT
ep PCR reverse primer (Δ*meq*)	GGGCGCTATGCCCTACAGTCCCGCTGACGATCCGTCCCCCCA GTCTACGGTCTGGTGGGCCAGTGTTACAACCAATTAACC
qPCR	
ICP4 forward primer	CGTGTTTTCCGGCATGTG
ICP4 reverse primer	TCCCATACCAATCCTCATCCA
ICP4 probe	FAM-CCCCCACCAGGTGCAGGCA-TAM
iNOS forward primer	GAGTGGTTTAAGGAGTTGGATCTGA
iNOS reverse primer	TTCCAGACCTCCCACCTCAA
iNOS probe	FAM-CTCTGCCTGCTGTTGCCAACATGC-TAM

[1]*en passant* mutagenesis.

FAM = 6-carboxyfluorescein; TAM = tetramethylrhodamine (TAMRA).
